# Effectiveness of the 23-Valent Pneumococcal Polysaccharide Vaccine (PPV23) in Preventing Community-Acquired Pneumonia in Adults: A Population-Based Cohort Study

**DOI:** 10.3390/vaccines12091023

**Published:** 2024-09-06

**Authors:** Mateu Serra-Prat, Ignasi Bolíbar, Elisabet Palomera, Àngel Lavado, Jordi Almirall

**Affiliations:** 1 Research Unit, Consorci Sanitari del Maresme, 08304 Mataró, Spain; epalomera@csdm.cat (E.P.); jalmirall@csdm.cat (J.A.); 2 Networked Biomedical Research Centre for Liver and Digestive Diseases (CIBEREHD), 28029 Madrid, Spain; 3 Department of Clinical Epidemiology and Public Health, Hospital de la Santa Creu i Sant Pau, 08025 Barcelona, Spain; ibolibar@santpau.cat; 4 Institute of Biomedical Research Sant Pau (IIB Sant Pau), 08035 Barcelona, Spain; 5 Department of Paediatrics, Obstetrics and Gynaecology and Preventive Medicine and Public Health, Universitat Autònoma de Barcelona, 08193 Bellaterra, Spain; 6 Networked Biomedical Research Centre for Epidemiology and Public Health (CIBERESP), 28029 Madrid, Spain; 7 Information Management Unit, Consorci Sanitari del Maresme, 08304 Mataró, Spain; alavado@csdm.cat; 8 Networked Biomedical Research Centre for Respiratory Diseases (CIBERES), 28029 Madrid, Spain

**Keywords:** community-acquired pneumonia, vaccination, PPV23, prevention, effectiveness

## Abstract

Aim: The aim was to assess the effectiveness of 23-valent pneumococcal polysaccharide vaccine (PPV23) in preventing CAP in adults. Methods: This was a population-based cohort study, followed up over 5 years (2015–2019), that included 47,768 persons aged ≥18 years assigned to three primary care centres. Data were retrospectively obtained from electronic medical records and databases. The vaccination effect was adjusted for potential confounders. Analyses were performed for the entire study population and for the ≥65 age subgroup. Results: The annual incidence of CAP (per 10^3^ adult inhabitants) was 3.29 overall, and 8.08 and 2.93 for vaccinated and non-vaccinated persons, respectively. The non-adjusted effect of PPV23 on CAP was evidenced by HR = 2.80 (95% CI: 2.32–3.37), and after adjusting for possible confounders, PPV23 showed no significant independent effect on CAP in the overall population (HR = 1.14; *p* = 0.277) or in persons aged ≥65 years (HR = 1.30; *p* = 0.051). No protective effect was observed in persons vaccinated <2 years previously (HR = 1.17; *p* = 0.514). Conclusions: PPV23 showed no effect in preventing CAP in adults aged ≥18 years or in the subgroup aged ≥65 years, even if vaccinated <2 years previously.

## 1. Introduction

Community-acquired pneumonia (CAP) is an acute respiratory infectious disease, acquired outside the hospital, which affects the distal part of the lung parenchyma. In the general adult population, its incidence ranges from 1.1 to 29.0 cases per 1000 person-years [1]. CAP is considered an important cause of morbimortality worldwide. *Streptococcus pneumoniae* is a major cause of CAP [2,3], although a 2016 US study in adults ≥65 years hospitalized with radiologically confirmed CAP detected *S. pneumoniae* by urine antigen in only 8.2% of cases [4]. Anti-pneumococcal vaccines, which have emerged as one of the main strategies for preventing CAP, can also help prevent invasive pneumococcal disease and in the fight against antibiotic resistance [5]. Different vaccines available against *S. pneumoniae* in adults have been recommended for immunosuppressed patients, subjects ≥60–65 years, and persons with other risk factors for CAP, such as chronic disease, alcoholism, and tobacco use [6]. The 23-valent pneumococcal polysaccharide vaccine (PPV23) includes the greatest number of serotypes and has been used for decades. However, it has the drawbacks that it does not generate immune memory, does not prevent antibody levels decreasing over time, and does not act on nasopharyngeal colonization [7]. Moreover, although PPV23 effectiveness in preventing invasive pneumococcal disease has been demonstrated, its effectiveness in preventing pneumonia is uncertain. Systematic reviews (SRs) and meta-analyses (MAs) assessing the effectiveness of PPV23 in preventing CAP found inconsistent evidence and are inconclusive [8,9,10,11,12,13]. The most recent MA, published in 2023, does not show a statistically significant effect of PPV23 in preventing CAP either in the population ≥16 years or in that of 65 to 75 years [10], so further research in this field is required. The objective of this study was to assess the real-world effectiveness of PPV23 in preventing CAP in adults.

## 2. Methodology

Our retrospective cohort study included the adult population (47,836 inhabitants aged ≥18 years) attended to in 3 primary care centres in the Maresme region (Barcelona, Spain) followed up over 5 years (2015–2019). Data according to ICD-10 and ATC codes were retrospectively collected from computerized primary care medical records, the pharmacy prescription withdrawal database, the regional hospital (Hospital de Mataró) computerized medical records, and the laboratory database. All CAP cases identified in the study population between 1 January 2015 and 31 December 2019 (5 years) according to the 76 predefined ICD-10 codes presented in the Supplementary Materials were considered. Study variables included sociodemographic and lifestyle factors (age, sex, current/ex-smoker, and alcoholism), comorbidities, therapeutic risk factors, vaccination with PPV23, and CAP during the study period (operative definitions of the study variables and their corresponding ICD-10 and/or ATC codes are presented in the Supplementary Materials).

The mean annual accumulated incidence of CAP and its 95% CI were calculated for the overall sample and by vaccination status and the CAP relative risk (RR). Also evaluated was the bivariate (non-adjusted) relationship between PPV23 vaccination and CAP, using hazard ratio (HR) values. The adjusted effect of PPV23 on CAP was evaluated using multiple Cox regression analysis. All baseline factors associated with both PPV23 and CAP were included in an initial multivariate model using the stepwise method, and any remaining significant variables and PPV23 were included in the final multivariate model. The same analyses were also run for the population subgroup aged ≥65 years and for the entire population considering the vaccination status in four categories according to the time since the last dose (not vaccinated, last dose >5 years, between 2 and 5 years, or <2 years). Statistical significance was established at *p* < 0.05. The study protocol was approved by the local ethics committee (reference CEIm CSdM 39/21).

## 3. Results

The study included 47,836 persons with a mean (SD) age of 48.0 (17.8) years, 51.0% women. The main comorbidities were cardiovascular diseases (23.8%), diabetes (5.8%), asthma (5.3%), and chronic bronchitis or chronic obstructive pulmonary disease (COPD) (4.4%); moreover, 11.3% of the sample were proton pump inhibitor (PPI) users and 6.9% had been previously vaccinated with PPV23. 

In the 5-year study period, there were 786 cases of CAP, representing a mean annual accumulated incidence of 3.29 new CAP cases/10^3^ adult inhabitants (95% CI: 3.06–3.51): 8.08 (95% CI: 6.73–9.43) and 2.93 (95% CI: 2.71–3.16) for vaccinated and non-vaccinated adults, respectively. The non-adjusted effect of PPV23 on CAP was evidenced by RR = 2.76 (95% CI: 2.29–3.32) and HR = 2.80 (95% CI: 2.32–3.37). Table 1 shows the variables associated with PPV23 (in OR terms) and non-adjusted and adjusted effects on CAP (in HR terms) in the adult population (≥18 years). After adjusting for all possible confounders, PPV23 had no significant effect on CAP (HR = 1.14; *p* = 0.277). When PPV23 exposure was analysed as a function of time since the last dose, the multivariate analysis showed no effect for vaccinated compared to non-vaccinated persons: not for vaccination <2 years previously (HR = 1.174; *p* = 0.514), vaccination 2–5 years previously (HR = 1.190; *p* = 0.530), or for vaccination >5 years previously (HR = 1.111; *p* = 0.422) (see Table 2). Finally, the subgroup analysis of persons aged ≥65 years showed unadjusted and adjusted effects of PPV23 on CAP of HR = 1.61 (*p* < 0.001) and HR=1.30 (*p* = 0.051), respectively (see Table 3).

## 4. Discussion

Our results indicate that PPV23 may not be effective in protecting the adult population against CAP in real life, and that this lack of effectiveness may also apply when the last dose was administered <2 years previously and in the population aged ≥65 years—results that agree with many observational studies. However, our results should be interpreted with caution and taking into account the limitations of the study discussed later: mainly, the quality of data registration and coding and the possibility of residual confounding after adjustment.

SRs and MAs published regarding PPV23 effectiveness and efficacy in preventing CAP have reported heterogenic and inconclusive results. Of four MAs of observational studies [9,10,11,12], only one reported a statistically significant protective pooled effect for PPV23 [9], while of five MAs of randomized controlled trials (RCTs) [8,9,11,12,13], three reported a significant pooled effect of PPV23 [9,11]. The 2023 SR-MA by Farrar et al. [10] included five observational studies assessing the effectiveness of PPV23 against PPV23-type pneumococcal pneumonia in individuals aged ≥16 years vaccinated <5 years previously. Only two studies observed a statistically significant effect, while the MA results did not show a significant effect for either the population ≥16 years (pooled OR = 0.82; *p* = 0.106) or for the population aged 65–74 years (pooled OR = 0.74; *p* = 0.095). Regarding the 2019 SR-MA by Winje et al. [11], of two RCTs assessing the efficacy of PPV23 in preventing CAP, one [14] reported a significant protective effect for nursing home residents (RR = 0.38; 95% CI: 0.21–0.69) [12], while the other [15] reported a non-significant effect for patients with COPD (RR = 0.09; 95% CI: 0.01–1.64). An MA of those two clinical trials [11] showed a significant protective effect of PPV23 in preventing CAP (RR = 0.36, i.e., risk reduction of 64%). However, the results of that MA were greatly influenced by the preponderant weight (95.8%) of the study that only included nursing home residents [14]. The Winje et al. SR-MA [11] also included two cohort studies [16,17] and three case–control studies [18,19,20] assessing PPV23 effectiveness in real-world conditions, with a significant protective effect reported for three of the five studies; however, the MA of those studies [11] did not show any statistically significant effect for either the cohort studies (OR = 0.76; 95% CI: 0.35–1.64) or the case–control studies (OR = 0.53; 95% CI: 0.28–1.01). The 2017 SR-MA by Falkenhorst et al. [9] reported pooled PPV23 efficacy against any pneumococcal pneumonia serotype of 64% (95% CI: 35–80%) for two clinical trials and effectiveness of 48% (95% CI: 25–63%) for two cohort studies. However, the 2016 SR-MA by Kraicer-Melamed [12], assessing PPV23 effectiveness in the general population ≥50 years, reported no significant effectiveness in preventing all-cause CAP, whether for clinical trials (4%; 95% CI: −26–26%), cohort studies (17%; 95% CI: −26–45%), or case–control studies (7%; 95% CI: −10–21%). Likewise, the 2016 SR-MA of RCTs by Schiffner-Rohe [13] also concluded that there was no proof that PPV23 can prevent pneumococcal pneumonia in the community-dwelling elderly population. Finally, the 2016 SR-MA by Diao et al. [8] that included seven RCTs concluded that PPV-23 provided weak protection against all-cause pneumonia in the immunocompetent population.

In summary, our results, similar to those of some other mentioned original and review studies, do not support the effectiveness of PPV23 in preventing CAP. This could be explained by the fact that not all pneumonias are bacterial, not all bacterial pneumonias are pneumococcal, and not all pneumococcal pneumonias are caused by the serotypes covered by PPSV23. Moreover, protection against certain serotypes can shift pneumonia to other serotypes against which an individual may have no immunity. In contrast, PPV23 effectiveness in preventing invasive pneumococcal disease seems much clearer, as MAs of observational studies report significant protective effects [10,11], justifying its use. Pneumococcal conjugate vaccines (PCVs)—such as PCV13 and the more recent PCV15 and PCV20—seem to show greater effectiveness. However, it would be of interest to assess and compare the short- and long-term effectiveness of these vaccines in relation to each other, especially considering that PCV15 and PCV20 were licenced in the US based only on safety and immunogenicity data.

This study has the limitations inherent to observational retrospective studies. First, since this population-based study is based on data recorded in electronic medical records and databases, its quality depends on data recording and coding accuracy and completeness. Misclassifications or under-recording of CAP episodes, vaccination status, or diseases or other potentially confounding variables may dilute the expected effect. We find no reason to think, if misclassification occurred, that it could have differed between the vaccinated and non-vaccinated subjects, i.e., misclassification may have diluted the expected effect, but not biased it. No data are available to quantify misclassification, so it is hard to estimate to what extent it may have influenced the studied vaccine effect. We think that the misclassification in the vaccine registration must be marginal, since this registration is essential and mandatory for its dispensing. The Spanish Society of Pulmonology and Thoracic Surgery has estimated that vaccination coverage against pneumococcus in Spain is 22% in the population ≥65 years [21,22]. In our study, we observed a coverage of 34.9% in this age group, which suggests no under-registration. On the other hand, the prevalence observed in the comorbidities that can act as confounding factors are more or less as expected, which does not indicate a too significant under-registration in most chronic conditions. However, we suspect that the registry of some acute diseases (such as periodontitis or upper respiratory tract infection) or other clinical conditions such as alcoholism is low. The main problem may be in the misclassification of CAP, considering CAP in a clinical condition that was not (for instance, COPD exacerbation, bronchitis, or other low respiratory tract infections). Misclassification is undoubtedly a threat to this type of study that has been little evaluated and forces a cautious interpretation. Second, although we adjusted for all possible confounders (age, comorbidities, tobacco use, corticosteroid treatment), some residual confounding cannot be ruled out, such as the severity of some chronic clinical conditions or the presence of some acute ones. As non-risk groups are not vaccinated, we repeated our analysis for the population aged ≥65 years—i.e., vaccinated on the basis of the age criterion—thereby homogenizing and improving comparability between the vaccinated and non-vaccinated groups; however, we observe no adjusted effect of PPV23 on CAP prevention in this age subgroup.

In conclusion, PPV23 did not show a protective effect against CAP in the adult population in the Maresme region, and this lack of protective effect was also observed in people vaccinated <2 years previously and in the population aged ≥65 years. After adjusting for possible confounders, no significant or relevant effect was observed. More studies assessing the effectiveness of pneumococcal vaccines for CAP prevention are required, especially of more recently available new vaccines and those in the development phase.

## Figures and Tables

**Table 1:** Variables associated with PPV23 and non-adjusted and adjusted effects on CAP in adult population (≥18 years).

Total *n* = 47,836	Registered Cases *n* (%)	Association with PPV23	Non-Adjusted Effect on CAP	Adjusted Effect on CAP
		OR (95% CI)	HR (95% CI)	HR (95% CI)
Age *	47,836 (100%)	1.16 (1.15–1.16)	1.02 (1.02–1.03)	1.01 (1.01–1.02)
Sex	47,836 (100%)	0.86 (0.80–0.92)	1.06 (0.92–1.22)	
Chronic bronchitis/COPD *	2096 (4.4%)	5.07 (4.56–5.64)	2.72 (2.17–3.40)	1.57 (1.23–2.01)
Asthma *	2555 (5.3%)	2.18 (1.93–2.46)	1.61 (1.25–2.08)	1.22 (0.94–1.59)
Functional impairment *	680 (1.4%)	20.6 (17.6–24.1)	2.61 (1.79–3.81)	0.98 (0.65–1.48)
Periodontitis	77 (0.2%)	1.14 (0.50–2.63)	0.79 (0.11–5.58)	
Immunosuppressor treatment	149 (0.3%)	0.67 (0.31–1.42)	2.07 (0.86–4.98)	
Corticosteroid treatment *	928 (1.9%)	2.69 (2.25–3.21)	2.09 (1.46–3.00)	1.30 (0.89–1.89)
Proton pump inhibitor use *	5427 (11.3%)	5.66 (5.24–6.12)	2.06 (1.73–2.45)	1.23 (1.01–1.48)
Malnutrition	2849 (6.0%)	1.22 (1.06–1.40)	1.05 (0.79–1.40)	
Previous CAP *	1155 (2.4%)	2.94 (2.56–3.38)	3.30 (2.69–4.04)	2.47 (1.99–3.06)
Alcoholism	70 (0.1%)	0.39 (0.10–1.62)	1.75 (0.44–7.00)	
Chronic heart disease *	839 (1.8%)	11.4 (9.85–13.1)	2.57 (1.82–3.62)	
Cardiovascular disease *	11,372 (23.8%)	18.9 (17.2–20.7)	2.34 (2.03–2.70)	1.30 (1.08–1.57)
Dysphagia *	236 (0.5%)	6.05 (4.58–8.00)	2.92 (1.61–5.29)	1.62 (0.89–2.96)
Active cancer *	1235 (2.6%)	6.69 (5.90–7.60)	2.37 (1.76–3.20)	1.28 (0.94–1.75)
Diabetes *	2773 (5.8%)	8.17 (7.47–8.94)	2.57 (2.09–3.15)	1.34 (1.07–1.68)
Upper respiratory tract infection *	577 (1.2%)	1.70 (1.31–2.21)	2.39 (1.57–3.65)	1.98 (1.29–3.03)
Chronic liver disease *	483 (1.0%)	2.44 (1.90–3.14)	2.06 (1.25–3.38)	1.22 (0.73–2.03)
Chronic neuropathy *	908 (1.9%)	12.2 (10.6–13.9)	2.89 (2.11–3.97)	1.33 (0.94–1.87)
HIV infection	81 (0.2%)	0.89 (0.36–2.20)	3.04 (1.14–8.11)	
Severe renal failure *	482 (1.0%)	16.0 (13.3–19.2)	1.93 (1.16–3.22)	0.77 (0.45–1.29)
Tobacco use *	2783 (5.8%)	1.44 (1.26–1.64)	1.37 (1.05–1.78)	1.22 (0.93–1.59)
Professional dust exposure	1099 (2.3%)	0.01 (0.002–0.09)	0.83 (0.50–1.38)	
Professional cold exposure	686 (1.4%)	0.02 (0.003–0.14)	2.72 (2.15–3.45)	
Vaccination with PPV23	3292 (6.9%)	-	2.80 (2.32–3.37)	1.14 (0.90–1.44)

* Variables associated with PPV23 and CAP (possible confounders) included in the multivariate model. Abbreviations: CAP, community-acquired pneumonia; COPD, chronic obstructive pulmonary disease; HIV, human immunodeficiency virus.

**Table 2:** Effect of PPV23 vaccine according to time since last dose in adult (≥18 years) population.

	Annual Incidence of CAP (Cases/10^3^ Inhabitants)	Non-Adjusted Effect on CAP HR (95% CI)	Adjusted Effect on CAP * HR (95% CI)
Non-vaccinated	3.0	1	1
Last PPV23 dose > 5 years ago	8.2	2.81 (2.28–3.48)	1.11 (0.86–1.44)
Last PPV23 dose 2–5 years ago	8.0	2.74 (1.62–4.66)	1.19 (0.69–2.05)
Last PPV23 dose < 2 years	7.0	2.41 (1.51–3.85)	1.17 (0.73–1.90)

CAP: community-acquired pneumonia; HR: hazard ratio; PPV23: 23-valent pneumococcal polysaccharide vaccine. * Effect of PPV23 adjusted by age, chronic obstructive pulmonary disease or chronic bronchitis, proton pump inhibitors use, previous CAP, cardiovascular diseases, diabetes and upper respiratory tract infection.

**Table 3:** Variables associated with PPV23 and non-adjusted and adjusted effects on CAP in population ≥65 years.

Total *n* = 9212	Registered Cases *n* (%)	Association with PPV23	Non-Adjusted Effect on CAP	Adjusted Effect on CAP
		OR (95% CI)	HR (95% CI)	HR (95% CI)
Age *	9212 (100%)	1.09 (1.08–1.09)	1.01 (0.997–1.02)	0.999 (0.98–1.01)
Sex	9212 (100%)	1.05 (0.96–1.15)	1.62 (1.28–2.05)	1.47 (1.15–1.88)
Chronic bronchitis/COPD *	991 (10.7%)	2.15 (1.88–2.45)	1.81 (1.33–2.46)	1.36 (0.99–1.86)
Asthma *	731 (7.9%)	1.93 (1.65–2.24)	1.94 (1.39–2.71)	-
Functional impairment	574 (6.2%)	4.37 (3.64–5.23)	1.48 (0.98–2.23)	-
Periodontitis	19 (0.2%)	0.86 (0.33–2.27)	1.71 (0.24–12.15)	-
Immunosuppressor treatment	33 (0.4%)	0.33 (0.13–0.86)	3.10 (0.99–9.66)	-
Corticosteroid treatment	317 (3.4%)	1.60 (1.28–2.01)	1.59 (0.94–2.67)	-
Proton pump inhibitor use	2684 (29.1%)	1.76 (1.60–1.93)	1.22 (0.95–1.56)	--
Malnutrition	459 (5.0%)	1.87 (1.55–2.26)	0.92 (0.53–1.61)	-
Previous CAP *	282 (3.1%)	2.24 (1.84–2.71)	2.45 (1.78–3.37)	2.03 (1.46–2.82)
Alcoholism	18 (0.2%)	0.23 (0.05–1.01)	3.77 (0.94–15.23)	-
Chronic heart disease *	640 (6.9%)	2.54 (2.16–2.99)	1.49 (1.01–2.20)	-
Cardiovascular disease *	5835 (63.3%)	4.12 (3.71–4.57)	1.64 (1.25–2.13)	1.36 (0.95–1.68)
Dysphagia *	138 (1.5%)	1.94 (1.39–2.72)	2.23 (1.15–4.33)	1.94 (0.99–3.79)
Active cancer *	804 (8.7%)	1.70 (1.47–1.96)	1.1.59 (1.13–2.25)	1.29 (0.91–1.83)
Diabetes *	1779 (19.3%)	2.04 (1.84–2.27)	1.61 (1.24–2.09)	1.32 (1.01–1.72)
Upper respiratory tract infection	147 (1.6%)	1.33 (0.96–1.85)	3.65 (2.17–6.14)	-
Chronic liver disease	186 (2.0%)	1.13 (0.84–1.52)	0.88 (0.36–2.13)	-
Chronic neuropathy *	719 (7.8%)	2.50 (2.14–2.92)	1.75 (1.23–2.48)	1.40 (0.97–2.02)
HIV infection	11 (0.1%)	1.54 (0.47–5.10)	3.09 (0.43–22.01)	-
Severe renal failure	406 (4.4%)	3.13 (2.55–3.84)	1.14 (0.66–1.95)	-
Tobacco use	531 (5.8%)	1.68 (1.41–2.00)	1.39 (0.90–2.15)	-
Vaccination with PPV23	3212 (34.9%)	---	1.61 (1.27–2.04)	1.30 (0.999–1.69)

* Variables associated with PPV23 and CAP (possible confounders) included in the multivariate model. Abbreviations: CAP, community-acquired pneumonia; COPD, chronic obstructive pulmonary disease; HIV, human immunodeficiency virus.

## Data Availability

The dataset from this study is held securely in a Consorci Sanitari del Maresme (CSdM) server. Access must be requested at mserra@csdm.cat.

## Supplementary Materials

The following supporting information can be downloaded at: https://www.mdpi.com/article/10.3390/vaccines12091023/s1, Operative definitions of study variables with their corresponding ICD-10 and/or ATC codes.
