# Supplementary material for: Effectiveness of the 23-Valent Pneumococcal Polysaccharide Vaccine (PPV23) in Preventing Community-Acquired Pneumonia in Adults: A Population-Based Cohort Study
Source: Vaccines (Basel). 2024 Sep 6;12(9):1023. doi: 10.3390/vaccines12091023 (PMC11435684; doi:10.3390/vaccines12091023)
Supplement: Supplementary file 1 [file vaccines-12-01023-s001.zip › vaccines-3145374-supplementary.pdf]

Pneumonia		Chronic bronchitis / Chronic Obstructive Pulmonary Disease	Asthma	Functional impairment	Malnutrition
B58.3	J15.7	J40	J45.20	Z73.6	R63.4
B59	J15.8	J41.0	J45.21	Z74.01	R63.6
B77.81	J15.9	J41.1	J45.22	Z74.09	E40
A01.03	J16	J41.8	J45.30	Z99.3	E41
A02.22	J16.0	J42	J45.31		E42
A15.0	J16.8	J43.1	J45.32		E43
A21.2	J17	J43.8	J45.40		E44.0
A22.1	J18	J43.9	J45.41		E44.1
A37.01	J18.0	J44.0	J45.42		E46
A37.1	J18.1	J44.1	J45.50		
A37.11	J18.2	J44.9	J45.51		
A37.81	J18.8		J45.52		
A37.91	J18.9		J45.901		
A42.0	A69.8		J45.902		
A43.0	B01.2		J45.909		
A48.1	B05.2		J45.990		
A54.84	B06.81		J45.991		
J09.X1	B25.0		J45.998		
J10.00	B37.1				
J10.01	B38.0				
J10.08	B38.1				
J11.00	B38.2				
J11.08	B39.0				
J12.0	B39.1				
J12.1	B39.2				
J12.2	B44.0				
J12.3	B44.1				
J12.8	J85.1				
J12.81	J95.81				
J12.82					
J12.89					
J12.9					
J13					
J14					
J15					
J15.0					
J15.1					
J15.2					
J15.20					
J15.21					
J15.211					
J15.212					
J15.29					
J15.3					
J15.4					
J15.5					
J15.6					

Alcohol abuse	Periodontitis					Congestive heart failure	
F10.232	K04.5	K06.020	K08.25	K04.01	K09.1	I05.0	Q22.3
F10.239	K04.6	K06.021	K08.26	K04.02	K09.8	I05.1	Q22.4
F10.24	K04.7	K06.022	K08.3	K04.1	K09.9	I05.2	Q22.5
F10.250	K04.8	K06.023	K08.4	K04.2		I05.8	Q22.6
F10.251	K04.9	K06.1	K08.40	K04.3		I05.9	Q22.8
F10.259	K04.90	K06.2	K08.401	K04.4		I06.0	Q22.9
F10.26	K04.99	K06.3	K08.402	K08.411		I06.1	Q23.0
F10.27	K05.0	K06.8	K08.403	K08.412		I06.2	Q23.1
F10.280	K05.00	K06.9	K08.404	K08.413		I06.8	Q23.2
F10.281	K05.01	K08.0	K08.409	K08.414		I06.9	Q23.3
F10.282	K05.1	K08.1	K08.41	K08.419		I07.0	Q23.4
F10.288	K05.10	K08.10	K00.0	K08.42		I07.1	Q23.8
F10.29	K05.11	K08.101	K00.1	K08.421		I07.2	Q23.9
F10.10	K05.2	K08.102	K00.2	K08.422		I07.8	I50.1
F10.11	K05.20	K08.103	K00.3	K08.423		I07.9	I50.20
F10.120	K05.21	K08.104	K00.4	K08.424		I08.0	I50.22
F10.121	K05.211	K08.109	K00.5	K08.429		I08.1	I50.23
F10.129	K05.212	K08.11	K00.6	K08.43		I08.2	I50.30
F10.14	K05.213	K08.111	K00.7	K08.431		I08.3	I50.32
F10.150	K05.219	K08.112	K00.8	K08.432		I08.8	I50.33
F10.151	K05.22	K08.113	K00.9	K08.433		I08.9	I50.40
F10.159	K05.221	K08.114	K01.0	K08.434		I09.1	I50.42
F10.180	K05.222	K08.119	K01.1	K08.439		I34.0	I50.43
F10.181	K05.223	K08.12	K02.3	K08.49		I34.1	I50.810
F10.182	K05.229	K08.121	K02.5	K08.491		I34.2	I50.812
F10.188	K05.3	K08.122	K02.51	K08.492		I34.8	I50.813
F10.19	K05.30	K08.123	K02.52	K08.493		I34.9	I50.814
F10.20	K05.31	K08.124	K02.53	K08.494		I35.0	I50.82
F10.21	K05.311	K08.129	K02.6	K08.499		I35.1	I50.83
F10.220	K05.312	K08.13	K02.61	K08.5		I35.2	I50.84
F10.221	K05.313	K08.131	K02.62	K08.50		I35.8	I50.89
F10.229	K05.319	K08.132	K02.63	K08.51		I35.9	I50.9
F10.230	K05.32	K08.133	K02.7	K08.52		I36.0	
F10.231	K05.321	K08.134	K02.9	K08.53		I36.1	
	K05.322	K08.139	K03.0	K08.530		I36.2	
	K05.323	K08.19	K03.1	K08.531		I36.8	
	K05.329	K08.191	K03.2	K08.539		I36.9	
	K05.4	K08.192	K03.3	K08.54		I37.0	
	K05.5	K08.193	K03.4	K08.55		I37.1	
	K05.6	K08.194	K03.5	K08.56		I37.2	
	K06.0	K08.199	K03.6	K08.59		I37.8	
	K06.01	K08.2	K03.7	K08.8		I37.9	
	K06.010	K08.20	K03.8	K08.81		I38	
	K06.011	K08.21	K03.81	K08.82		I39	
	K06.012	K08.22	K03.89	K08.89		Q22.0	
	K06.013	K08.23	K03.9	K08.9		Q22.1	
	K06.02	K08.24	K04.0	K09.0		Q22.2	

Dysphagia	Upper Respiratory Track Infection	Chronic Liver Disease		Chronic Kidney Disease		HIV
K22.4	J00	K70.0	K76.5	N00.0	N04.7	B97.35
D50.1	J01.00	K70.10	K76.6	N00.1	N04.8	Z21
I69.091	J01.01	K70.11	K76.7	N00.2	N04.9	B20
I69.191	J01.10	K70.2	K76.81	N00.3	N05.0	
I69.291	J01.11	K70.30	K76.89	N00.4	N05.1	
I69.391	J01.20	K70.31	K76.9	N00.5	N05.2	
I69.891	J01.21	K70.40		N00.6	N05.3	
I69.991	J01.30	K70.41		N00.7	N05.4	
R13.10	J01.31	K70.9		N00.8	N05.5	
R13.11	J01.40	K71.0		N00.9	N05.6	
R13.12	J01.41	K71.10		N01.0	N05.7	
R13.13	J01.80	K71.11		N01.1	N05.8	
R13.14	J01.81	K71.2		N01.2	N05.9	
R13.19	J01.90	K71.3		N01.3	N06.0	
	J01.91	K71.4		N01.4	N06.1	
	J02.0	K71.50		N01.5	N06.2	
	J02.8	K71.51		N01.6	N06.3	
	J02.9	K71.6		N01.7	N06.4	
	J03.00	K71.7		N01.8	N06.5	
	J03.01	K71.8		N01.9	N06.6	
	J03.80	K71.9		N02.0	N06.7	
	J03.81	K72.10		N02.1	N06.8	
	J03.90	K72.11		N02.2	N06.9	
	J03.91	K72.90		N02.3	N07.0	
	J04.0	K72.91		N02.4	N07.1	
	J04.10	K73.0		N02.5	N07.2	
	J04.11	K73.1		N02.6	N07.3	
	J04.2	K73.2		N02.7	N07.4	
	J04.30	K73.8		N02.8	N07.5	
	J04.31	K73.9		N02.9	N07.6	
	J05.0	K74.0		N03.0	N07.7	
	J05.10	K74.1		N03.1	N07.8	
	J05.11	K74.2		N03.2	N07.9	
	J06.0	K74.3		N03.3	N08	
	J06.9	K74.4		N03.4	N18.4	
	J30.0	K74.5		N03.5	N18.5	
	J30.1	K74.60		N03.6	N18.6	
	J30.2	K74.69		N03.7	N18.9	
	J30.5	K75.0		N03.8		
	J30.81	K75.1		N03.9		
	J30.89	K75.3		N04.0		
	J30.9	K75.4		N04.1		
	J31.0	K75.89		N04.2		
	J31.1	K75.9		N04.3		
	J31.2	K76.1		N04.4		
		K76.2		N04.5		
		K76.3		N04.6		

Cancer							
C10.2	C18.7	C91.02	C43.0	C50.629	C75.9	C81.04	C31.8
C10.3	C18.8	C91.10	C43.10	C50.811	C76.0	C81.05	C31.9
C10.4	C18.9	C91.11	C43.111	C50.812	C76.1	C81.06	C32.0
C10.8	C19	C91.12	C43.112	C50.819	C76.2	C81.07	C32.1
C10.9	C20	C91.30	C43.121	C50.821	C76.3	C81.08	C32.2
C11.0	C21.0	C91.31	C43.122	C50.822	C76.40	C81.09	C32.3
C11.1	C21.1	C91.32	C43.20	C50.829	C76.41	C81.10	C32.8
C11.2	C21.2	C91.40	C43.21	C50.911	C76.42	C81.11	C32.9
C11.3	C21.8	C91.41	C43.22	C50.912	C76.50	C81.12	C33
C11.8	C22.0	C91.42	C43.30	C50.919	C76.51	C81.13	C34.00
C11.9	C22.1	C91.50	C43.31	C50.921	C76.52	C81.14	C34.01
C12	C22.2	C91.51	C43.39	C50.922	C76.8	C81.15	C34.02
C13.0	C22.3	C91.52	C43.4	C50.929	C77.0	C81.16	C84.A0
C13.1	C85.93	C91.60	C43.51	C51.0	C77.1	C81.17	C84.A1
C13.2	C85.94	C91.61	C43.52	C51.1	C77.2	C81.18	C84.A2
C13.8	C85.95	C91.62	C43.59	C51.2	C77.3	C81.19	C84.A3
C13.9	C85.96	C91.90	C43.60	C51.8	C77.4	C81.20	C84.A4
C14.0	C85.97	C91.91	C43.61	C72.1	C77.5	C81.21	C84.A5
C14.2	C85.98	C91.92	C43.62	C72.20	C77.8	C81.22	C84.A6
C14.8	C85.99	C38.4	C43.70	C72.21	C77.9	C81.23	C84.A7
C15.3	C86.0	C38.8	C50.219	C72.22	C78.00	C81.24	C84.A8
C15.4	C86.1	C39.0	C50.221	C72.30	C78.01	C22.4	C84.A9
C15.5	C86.2	C39.9	C50.222	C72.31	C7B.01	C22.7	C84.Z0
C15.8	C86.3	C40.00	C50.229	C72.32	D46.0	C22.8	C84.Z1
C15.9	C86.4	C40.01	C50.311	C72.40	D46.1	C22.9	C84.Z2
C16.0	C86.5	C40.02	C50.312	C72.41	D46.20	C23	C84.Z3
C16.1	C86.6	C40.10	C50.319	C72.42	D46.21	C24.0	C84.Z4
C16.2	C88.0	C40.11	C50.321	C72.50	D46.22	C24.1	C91.A0
C16.3	C88.2	C40.12	C50.322	C72.59	D46.4	C24.8	C91.A1
C16.4	C88.3	C40.20	C50.329	C72.9	D46.9	C24.9	C91.A2
C16.5	C88.4	C40.21	C50.411	C73	D46.A	C25.0	C91.Z0
C16.6	C88.8	C40.22	C50.412	C74.00	D46.B	C25.1	C91.Z1
C16.8	C88.9	C40.30	C50.419	C74.01	D46.C	C25.2	C91.Z2
C16.9	C90.00	C40.31	C50.421	C74.02	D46.Z	C25.3	C92.00
C17.0	C90.01	C40.32	C50.422	C74.10	C7B.02	C25.4	C92.01
C17.1	C90.02	C40.80	C50.429	C74.11	C7B.03	C25.7	C92.02
C17.2	C90.10	C40.81	C50.511	C74.12	C7B.04	C25.8	C92.10
C17.3	C90.11	C40.82	C50.512	C74.90	C7B.09	C25.9	C92.11
C17.8	C90.12	C40.90	C50.519	C74.91	C7B.1	C26.0	C92.12
C17.9	C90.20	C40.91	C50.521	C74.92	C7B.8	C26.1	C92.20
C18.0	C90.21	C40.92	C50.522	C75.0	C80.0	C26.9	C92.21
C18.1	C90.22	C41.0	C50.529	C75.1	C80.1	C30.0	C92.22
C18.2	C90.30	C41.1	C50.611	C75.2	C80.2	C30.1	C92.30
C18.3	C90.31	C41.2	C50.612	C75.3	C81.00	C31.0	C92.31
C18.4	C90.32	C41.3	C50.619	C75.4	C81.01	C31.1	C92.32
C18.5	C91.00	C41.4	C50.621	C75.5	C81.02	C31.2	C92.40
C18.6	C91.01	C41.9	C50.622	C75.8	C81.03	C31.3	C92.41

Cancer							
C92.42	C46.50	C56.1	C78.1	C7A.091	C81.92	C82.93	C83.70
C92.50	C46.51	C56.2	C78.2	C7A.092	C81.93	C82.94	C83.71
C92.51	C46.52	C56.9	C78.30	C7A.093	C81.94	C82.95	C83.72
C92.52	C46.7	C57.00	C78.39	C7A.094	C81.95	C82.96	C83.73
C92.60	C46.9	C57.01	C78.4	C7A.095	C82.40	C82.97	C83.74
C92.61	C47.0	C57.02	C78.5	C7A.096	C82.41	C82.98	C83.75
C92.62	C47.10	C57.10	C78.6	C7A.098	C82.42	C82.99	C83.76
C92.90	C47.11	C57.11	C78.7	C7A.1	C82.43	C83.00	C83.77
C92.91	C47.12	C57.12	C78.80	C7A.8	C82.44	C83.01	C83.78
C92.92	C47.20	C57.20	C78.89	C7B.00	C82.45	C83.02	C83.79
C92.A0	C47.21	C57.21	C79.00	C81.25	C82.46	C83.03	C83.80
C92.A1	C47.22	C57.22	C79.01	C81.26	C82.47	C83.04	C83.81
C92.A2	C47.3	C57.3	C79.02	C81.27	C82.48	C83.05	C83.82
C92.Z0	C47.4	C57.4	C79.10	C81.28	C82.49	C83.06	C83.83
C92.Z1	C47.5	C57.7	C79.11	C81.29	C82.50	C83.07	C83.84
C92.Z2	C47.6	C57.8	C79.19	C81.30	C82.51	C83.08	C83.85
C93.00	C47.8	C57.9	C79.2	C81.31	C82.52	C83.09	C83.86
C93.01	C47.9	C58	C79.31	C81.32	C82.53	C83.10	C83.87
C93.02	C48.0	C60.0	C79.32	C81.33	C82.54	C83.11	C83.88
C93.10	C48.1	C60.1	C79.40	C81.34	C82.55	C83.12	C83.89
C93.11	C48.2	C60.2	C79.49	C81.35	C82.56	C83.13	C83.90
C93.12	C48.8	C60.8	C79.51	C81.36	C82.57	C83.14	C83.91
C93.30	C49.0	C60.9	C79.52	C81.37	C82.58	C83.15	C83.92
C93.31	C49.10	C61	C79.60	C81.38	C82.59	C83.16	C83.93
C93.32	C49.11	C62.00	C79.61	C81.39	C82.60	C83.17	C83.94
C93.90	C49.12	C62.01	C79.62	C81.40	C82.61	C83.18	C83.95
C93.91	C49.20	C62.02	C79.70	C81.41	C82.62	C83.19	C83.96
C93.92	C49.21	C62.10	C79.71	C81.42	C82.63	C83.30	C83.97
C93.Z0	C49.22	C62.11	C79.72	C81.43	C82.64	C83.31	C83.98
C93.Z1	C49.3	C62.12	C79.81	C81.44	C82.65	C83.32	C83.99
C93.Z2	C49.4	C62.90	C79.82	C81.45	C82.66	C83.33	C84.00
C94.00	C49.5	C62.91	C79.89	C81.46	C82.67	C83.34	C84.01
C94.01	C49.6	C62.92	C79.9	C81.47	C82.68	C83.35	C84.02
C43.71	C49.8	C63.00	C7A.00	C81.48	C82.69	C83.36	C84.03
C43.72	C51.9	C63.01	C7A.010	C81.49	C82.80	C83.37	C84.04
C43.8	C52	C63.02	C7A.011	C81.70	C82.81	C83.38	C84.05
C43.9	C53.0	C63.10	C7A.012	C81.71	C82.82	C83.39	C84.06
C45.0	C53.1	C63.11	C7A.019	C81.72	C82.83	C83.50	C84.07
C45.1	C53.8	C63.12	C7A.020	C81.73	C82.84	C83.51	C84.08
C45.2	C53.9	C63.2	C7A.021	C81.74	C82.85	C83.52	C84.09
C45.7	C54.0	C63.7	C7A.022	C81.75	C82.86	C83.53	C84.10
C45.9	C54.1	C63.8	C7A.023	C81.76	C82.87	C83.54	C84.11
C46.0	C54.2	C63.9	C7A.024	C81.77	C82.88	C83.55	C84.12
C46.1	C54.3	C64.1	C7A.025	C81.78	C82.89	C83.56	C84.13
C46.2	C54.8	C64.2	C7A.026	C81.79	C82.90	C83.57	C84.14
C46.3	C54.9	C64.9	C7A.029	C81.90	C82.91	C83.58	C84.15
C46.4	C55	C78.02	C7A.090	C81.91	C82.92	C83.59	C84.16

Cancer						
C84.17	C02.9	C85.23	C96.29	C4A.52	C82.19	C69.20
C84.18	C03.0	C85.24	C96.4	C4A.59	C82.20	C69.21
C84.19	C03.1	C85.25	C96.5	C4A.60	C82.21	C69.22
C84.40	C03.9	C85.26	C96.6	C4A.61	C82.22	C69.30
C84.41	C04.0	C85.27	C96.9	C4A.62	C82.23	C69.31
C84.42	C04.1	C85.28	C96.A	C4A.70	C82.24	C69.32
C84.43	C04.8	C85.29	C96.Z	C4A.71	C82.25	C69.40
C84.44	C04.9	C85.80	C34.10	C4A.72	C82.26	C69.41
C84.45	C05.0	C85.81	C34.11	C4A.8	C82.27	C69.42
C84.46	C05.1	C85.82	C34.12	C4A.9	C82.28	C69.50
C84.47	C05.2	C85.83	C34.2	C50.011	C82.29	C69.51
C84.48	C05.8	C85.84	C34.30	C50.012	C82.30	C69.52
C84.49	C05.9	C85.85	C34.31	C50.019	C82.31	C69.60
C84.60	C06.0	C85.86	C34.32	C50.021	C82.32	C69.61
C84.61	C06.1	C85.87	C34.80	C50.022	C82.33	C69.62
C84.62	C06.2	C85.88	C34.81	C50.029	C82.34	C69.80
C84.63	C06.80	C85.89	C34.82	C50.111	C82.35	C69.81
C84.64	C06.89	C85.90	C34.90	C50.112	C82.36	C69.82
C84.65	C06.9	C85.91	C34.91	C50.119	C82.37	C69.90
C84.66	C07	C85.92	C34.92	C50.121	C82.38	C69.91
C84.67	C08.0	C94.02	C37	C50.122	C82.39	C69.92
C84.68	C08.1	C94.20	C38.0	C50.129	C65.1	C70.0
C84.69	C08.9	C94.21	C38.1	C50.211	C65.2	C70.1
C84.70	C09.0	C94.22	C38.2	C50.212	C65.9	C70.9
C84.71	C09.1	C94.30	C38.3	C81.96	C66.1	C71.0
C84.72	C09.8	C94.31	C49.9	C81.97	C66.2	C71.1
C84.73	C09.9	C94.32	C49.A0	C81.98	C66.9	C71.2
C84.74	C10.0	C94.40	C49.A1	C81.99	C67.0	C71.3
C84.75	C10.1	C94.41	C49.A2	C82.00	C67.1	C71.4
C84.76	C84.Z5	C94.42	C49.A3	C82.01	C67.2	C71.5
C84.77	C84.Z6	C94.6	C49.A4	C82.02	C67.3	C71.6
C00.0	C84.Z7	C94.80	C49.A5	C82.03	C67.4	C71.7
C00.1	C84.Z8	C94.81	C49.A9	C82.04	C67.5	C71.8
C00.2	C84.Z9	C94.82	C4A.0	C82.05	C67.6	C71.9
C00.3	C85.10	C95.00	C4A.10	C82.06	C67.7	C72.0
C00.4	C85.11	C95.01	C4A.111	C82.07	C67.8	C84.78
C00.5	C85.12	C95.02	C4A.112	C82.08	C67.9	C84.79
C00.6	C85.13	C95.10	C4A.121	C82.09	C68.0	C84.90
C00.8	C85.14	C95.11	C4A.122	C82.10	C68.1	C84.91
C00.9	C85.15	C95.12	C4A.20	C82.11	C68.8	C84.92
C01	C85.16	C95.90	C4A.21	C82.12	C68.9	C84.93
C02.0	C85.17	C95.91	C4A.22	C82.13	C69.00	C84.94
C02.1	C85.18	C95.92	C4A.30	C82.14	C69.01	C84.95
C02.2	C85.19	C96.0	C4A.31	C82.15	C69.02	C84.96
C02.3	C85.20	C96.20	C4A.39	C82.16	C69.10	C84.97
C02.4	C85.21	C96.21	C4A.4	C82.17	C69.11	C84.98
C02.8	C85.22	C96.22	C4A.51	C82.18	C69.12	C84.99

Diabetes Mellitus

E10.3393	E10.52	E11.3511	E11.641	E13.3531	E08.10	E08.3551
E10.3399	E10.59	E11.3512	E11.649	E13.3532	E08.11	E08.3552
E10.3411	E10.610	E11.3513	E11.65	E13.3533	E08.21	E08.3553
E10.3412	E10.618	E11.3519	E11.69	E13.3539	E08.22	E08.3559
E10.3413	E10.620	E11.3521	E11.8	E13.3541	E08.29	E08.3591
E10.3419	E10.621	E11.3522	E11.9	E13.3542	E08.311	E08.3592
E10.3491	E10.622	E11.3523	E13.00	E13.3543	E08.319	E08.3593
E10.3492	E10.628	E11.3529	E13.01	E13.3549	E08.3211	E08.3599
E10.3493	E10.630	E11.3531	E13.10	E13.3551	E08.3212	E08.36
E10.3499	E10.638	E11.3532	E13.11	E13.3552	E08.3213	E08.37X1
E10.3511	E10.641	E11.3533	E13.21	E13.3553	E08.3219	E08.37X2
E10.3512	E10.649	E11.3539	E13.22	E13.3559	E08.3291	E08.37X3
E10.3513	E10.65	E11.3541	E13.29	E13.3591	E08.3292	E08.37X9
E10.3519	E10.69	E11.3542	E13.311	E13.3592	E08.3293	E08.39
E10.3521	E10.8	E11.3543	E13.319	E13.3593	E08.3299	E08.40
E10.3522	E10.9	E11.3549	E13.3211	E13.3599	E08.3311	E08.41
E10.3523	E11.00	E11.3551	E13.3212	E13.36	E08.3312	E08.42
E10.3529	E11.01	E11.3552	E13.3213	E13.37X1	E08.3313	E08.43
E10.3531	E11.21	E11.3553	E13.3219	E13.37X2	E08.3319	E08.44
E10.3532	E11.22	E11.3559	E13.3291	E13.37X3	E08.3391	E08.49
E10.3533	E11.29	E11.3591	E13.3292	E13.37X9	E08.3392	E08.51
E10.3539	E11.311	E11.3592	E13.3293	E13.39	E08.3393	E08.52
E10.3541	E11.319	E11.3593	E13.3299	E13.40	E08.3399	E08.59
E10.3542	E11.3211	E11.3599	E13.3311	E13.41	E08.3411	E08.610
E10.3543	E11.3212	E11.36	E13.3312	E13.42	E08.3412	E08.618
E10.3549	E11.3213	E11.37X1	E13.3313	E13.43	E08.3413	E08.620
E10.3551	E11.3219	E11.37X2	E13.3319	E13.44	E08.3419	E08.621
E10.3552	E11.3291	E11.37X3	E13.3391	E13.49	E08.3491	E08.622
E10.3553	E11.3292	E11.37X9	E13.3392	E13.51	E08.3492	E08.628
E10.3559	E11.3293	E11.39	E13.3393	E13.52	E08.3493	E08.630
E10.3591	E11.3299	E11.40	E13.3399	E13.59	E08.3499	E08.638
E10.3592	E11.3311	E11.41	E13.3411	E13.610	E08.3511	E08.641
E10.3593	E11.3312	E11.42	E13.3412	E13.618	E08.3512	E08.649
E10.3599	E11.3313	E11.43	E13.3413	E13.620	E08.3513	E08.65
E10.36	E11.3319	E11.44	E13.3419	E13.621	E08.3519	E08.69
E10.37X1	E11.3391	E11.49	E13.3491	E13.622	E08.3521	E08.8
E10.37X2	E11.3392	E11.51	E13.3492	E13.628	E08.3522	E08.9
E10.37X3	E11.3393	E11.52	E13.3493	E13.630	E08.3523	E10.10
E10.37X9	E11.3399	E11.59	E13.3499	E13.638	E08.3529	E10.11
E10.39	E11.3411	E11.610	E13.3511	E13.641	E08.3531	E10.21
E10.40	E11.3412	E11.618	E13.3512	E13.649	E08.3532	E10.22
E10.41	E11.3413	E11.620	E13.3513	E13.65	E08.3533	E10.29
E10.42	E11.3419	E11.621	E13.3519	E13.69	E08.3539	E10.311
E10.43	E11.3491	E11.622	E13.3521	E13.8	E08.3541	E10.319
E10.44	E11.3492	E11.628	E13.3522	E13.9	E08.3542	E10.3211
E10.49	E11.3493	E11.630	E13.3523	E08.00	E08.3543	E10.3212
E10.51	E11.3499	E11.638	E13.3529	E08.01	E08.3549	E10.3213

Diabetes Mellitus	Immunosuppressors ATC	Oral corticosteroids ATC	PPI ATC
E10.3219	L04AA01	H02AA02	A02BC01
E10.3291	L04AA05	H02AB01	A02BC02
E10.3292	L04AA06	H02AB02	A02BC03
E10.3293	L04AA10	H02AB04	A02BC04
E10.3299	L04AA13	H02AB05	A02BC05
E10.3311	L04AA18	H02AB06	A02BC06
E10.3312	L04AA21	H02AB07	
E10.3313	L04AA33	H02AB08	
E10.3319	L04AA37	H02AB09	
E10.3391	L04AA44	H02AB10	
E10.3392	L04AA56	H02AB13	
	L04AB01		
	L04AB04	**ICD-10**	
	L04AC05	Z79.51	
	L04AC18	Z79.52	
	L04AC24		
	L04AD01		
	L04AD02		
	L04AD03		
	L04AX01		
	L04AX03		
	L04AX07		
